# Mapping of quantitative adult plant field resistance to leaf rust and stripe rust in two European winter wheat populations reveals co-location of three QTL conferring resistance to both rust pathogens

**DOI:** 10.1007/s00122-014-2357-0

**Published:** 2014-08-12

**Authors:** Maria Buerstmayr, Lydia Matiasch, Fabio Mascher, Gyula Vida, Marianna Ittu, Olivier Robert, Sarah Holdgate, Kerstin Flath, Anton Neumayer, Hermann Buerstmayr

**Affiliations:** 1 Department for Agrobiotechnology Tulln, BOKU-University of Natural Resources and Life Sciences Vienna, Konrad Lorenz Str. 20, Tulln, 3430 Austria; 2 Agroscope Changins-Wädenswil Research Station ACW, 1260 Nyon, Switzerland; 3 Agricultural Research Institute of the Hungarian Academy of Sciences, Martonvásár, 2462 Hungary; 4 National Agricultural Research Development Institute Fundulea, 915200 Fundulea, Romania; 5 Bioplante, 3 Rue Florimond Desprez, BP41, 59242 Cappelle-en- Pévèle, France; 6 RAGT Seeds, Grange Road, Ickleton, Essex, CB10 1TA UK; 7 Julius Kühn Institute, Federal Research Centre for Cultivated Plants, 14532 Kleinmachnow, Germany; 8 SAATZUCHT DONAU GmbH & CoKG, Reichersberg, 4981 Austria; 9 Present Address: NIAB, Huntingdon Road, Cambridge, CB3 0LE UK

## Abstract

*****Key message***:**

**We detected several, most likely novel QTL for adult plant resistance to rusts. Notably three QTL improved resistance to leaf rust and stripe rust simultaneously indicating broad spectrum resistance QTL.**

***Abstract*:**

The rusts of wheat (*Puccinia* spp.) are destructive fungal wheat diseases. The deployment of resistant cultivars plays a central role in integrated rust disease management. Durability of resistance would be preferred, but is difficult to analyse. The Austrian winter wheat cultivar Capo was released in the 1989 and grown on a large acreage during more than two decades and maintained a good level of quantitative leaf rust and stripe rust resistance. Two bi-parental mapping populations: Capo × Arina and Capo × Furore were tested in multiple environments for severity of leaf rust and stripe rust at the adult plant stage in replicated field experiments. Quantitative trait loci associated with leaf rust and stripe rust severity were mapped using DArT and SSR markers. Five QTL were detected in multiple environments associated with resistance to leaf rust designated as *QLr.ifa*-*2AL, QLr.ifa*-*2BL, QLr.ifa*-*2BS, QLr.ifa*-*3BS*, and *QLr.ifa*-*5BL*, and five for resistance to stripe rust *QYr.ifa*-*2AL, QYr.ifa*-*2BL, QYr.ifa*-*3AS, QYr.ifa*-*3BS*, and *QYr.ifa*-*5A*. For all QTL apart from two (*QYr.ifa*-*3AS, QLr.ifa*-*5BL*) Capo contributed the resistance improving allele. The leaf rust and stripe rust resistance QTL on 2AL, 2BL and 3BS mapped to the same chromosome positions, indicating either closely linked genes or pleiotropic gene action. These three multiple disease resistance QTL (*QLr.ifa*-*2AL/QYr.ifa*-*2AL*, *QLr.ifa.2BL/QYr.ifa*-*2BL*, *QLr.ifa*-*3BS/QYr.ifa.3BS*) potentially contribute novel resistance sources for stripe rust and leaf rust. The long-lasting resistance of Capo apparently rests upon a combination of several genes. The described germplasm, QTL and markers are applicable for simultaneous resistance improvement against leaf rust and stripe rust.

**Electronic supplementary material:**

The online version of this article (doi:10.1007/s00122-014-2357-0) contains supplementary material, which is available to authorized users.

## Introduction

Leaf rust (brown rust, Lr) and stripe rust (yellow rust, Yr), caused by *Puccinia triticina* and *P. striiformis* f. sp. *tritici*, respectively, are major biotic threats in many wheat-growing regions of the world. Genetic control of rust diseases offers a cost-effective and environmental-friendly strategy to reduce losses in wheat. Resistance to rust diseases is either quantitative (horizontal, uniform, race-non-specific, stable, adult plant resistance) or qualitative (vertical, differential, race-specific, unstable, seedling resistance) (Van der Plank 1963, 1968). Generally, race-specific resistances are governed by major genes, which confer complete resistance and are highly effective through the entire life cycle of the host plants. These genes usually initiate a hypersensitive response, leading to a rapid cell death upon infection by a pathogen race that carries a matching avirulence gene (Flor 1956; Heath 2000). Rust pathogens are notorious for their rapid adaptation to such genes. Consequently, cultivars that rely on race-specific genes may become susceptible within a few years (Priyamvada et al. 2011; Kolmer 2013). Stripe rust pathotypes with complex virulence profiles, increased aggressiveness and extended adaption to previously unfavorable environments have evolved (Hovmøller 2008; Milus 2009).

Currently, more than 70 formally and ten temporarily designated *Lr* genes and more than 50 formally and around 40 temporarily designated *Yr* genes have been described (McIntosh et al. 2012). Most of these genes confer race-specific resistance (Lin and Chen 2007; Bolton 2008). Only a few of these extensively used genes remained effective over a long period (Singh et al. 1997; Chen 2007; Lowe 2011a; Ren et al. 2012). In contrast to race-specific resistance genes, the combination of several partial, race-non-specific resistances constitutes an alternative by providing more durable resistance. These genes usually contribute incomplete, minor to intermediate quantitative, partial resistance due to reduced receptivity, increased latent period, smaller and fewer uredinia (Ohm and Shaner 1976; Parlevliet 1985). Partial resistance genes are generally more effective at adult plant stages and are also termed adult plant resistance (APR) genes, although not all APR genes are race-non-specific (McCallum et al. 2012). Breeding for durable resistance is an important but difficult task. Johnson (1981) described durable resistance as resistance that remains effective when deployed over an extensive acreage and time in an environment favorable for the disease. Therefore, durability can be confirmed only retrospectively over the course of time. Three rust resistance gene complexes are in accordance with the definition of durable resistance, *Lr34/Yr18/Pm38* (McIntosh 1992; Spielmeyer et al. 2005; Krattinger et al. 2009; Lagudah et al. 2009), *Lr46/Yr29/Pm39* (Singh et al. 1998; William et al. 2003; Lillemo et al. 2008) and *Sr2/Yr30* (McIntosh 1995; Singh et al. 2001; Rehman et al.^2013^). All together they confer non-hypersensitive, slow-rusting, partial type of resistance. Partial resistance genes are of great interest as sources of potentially more durable resistance, although not all partial resistance genes are durable (Johnson 1992). This has initiated a number of QTL studies, which have reported the detection of numerous QTL. Rosewarne et al. (2013) provides an update review about QTL for stripe rust in wheat published during the last 10 years, summarizing more than 140 QTL assigned to 49 chromosomal locations. Although many of these QTL may be redundant, it illustrates a great genetic diversity for this trait. Partial resistance genes have also been found for leaf rust. To date, more than 20 QTL have been described (William et al.^1997^; ^2006^; Nelson et al. 1997; Faris et al. 1999; Messmer et al. 2000; Suenaga et al. 2003; Schnurbusch et al. 2004; Navabi et al. 2005; Xu et al. 2005a, b; Rosewarne et al. 2008; Maccaferri et al. 2008; 2010; Li et al. 2009; Chu et al. 2009; Singh et al. 2009). Partial resistance genes are additive in their effect and lead to enhanced resistance when combined. Under high disease pressure a combination of 4–5 such genes is required to provide sufficiently high levels of resistance (Singh et al. 2000a; Singh et al. 2011a). The Austrian winter wheat cultivar Capo was released in1989 and has been grown on a large-acreage in Austria, Hungary, Rumania and Slovakia, as well as Germany and France since then. Taken together, Capo has been grown on more than one Million ha since its release. And it is still the most important bread-making quality winter wheat in Austria (BAES 2013). Capo is a good yielding, high quality winter wheat with medium to good resistance to various diseases. It combines high resistance to stripe rust with moderate resistance to leaf rust that has remained stable since it was released. Previous screenings for resistance to leaf rust rated Capo susceptible at the seedling stage, but resistant at adult plant stage in field tests, indicating that it carries effective APR (Winzeler et al. 2000). Similarly, Pathan and Park (2006) verified the presence of unknown APR *Lr* gene(s) in addition to the seedling resistance gene *Lr13.* Multi-pathotype analysis for stripe rust identified seedling resistance gene *Yr27* and additional unknown seedling resistance gene(s) (Pathan et al. 2008). Our study uses two independent mapping populations to characterize the genetics of resistance of Capo to both leaf rust and stripe rust at the adult plant stage.

## Materials and methods

### Plant material

Two mapping populations comprising 233 and 201 recombinant inbred lines (RILs) were developed by single seed descent from crosses of Capo and Arina (CA) and of Capo and Furore (CF), respectively. Capo (Diplomat/Purdue5517//Extrem/HP3517 (= Pokal/Martin)) is a high quality winter wheat cultivar developed by Probstdorfer Saatzucht, Austria. Arina (Moisson/Zenith) is a high quality winter wheat cultivar developed by Agroscope, Switzerland and was released in 1981. Arina is highly susceptible in field tests to leaf rust and susceptible to stripe rust. Furore (Carolus//Pokal/Martin (one parent is a sister line of Capo)) is a quality winter wheat cultivar developed by Probstdorfer Saatzucht, Austria. It is moderately to highly susceptible to leaf rust and highly susceptible to stripe rust.

Multi-pathotype screening at seedling stage with a set of 20 different pathotypes of *P. triticina*, which were in total virulent to *Lr1*, *Lr2a*, *Lr2c*, *Lr3a*, *Lr3bg*, *Lr3ka*, *10*, *Lr11*, *Lr13*, *Lr 4a*, *Lr 5*, *Lr 6*, *Lr17a*, *Lr17b*, *Lr20*, *Lr23*, *Lr26*, *Lr27* + *Lr31*, postulated *Lr13* for both, Capo and Arina (Park et al. 2001). Field tests using either pathotypes virulent against genes *Lr1, Lr2c, Lr3a, Lr3bg, Lr3ka, Lr10, Lr13, Lr14a, Lr16, Lr17b, Lr20, Lr23, Lr24, Lr26* (Pathan and Park 2006) or local races of unknown pathogenicity (Winzeler et al. 2000) ascertained high adult plant field resistance for Capo and susceptibility for Arina. Capo and Arina were screened for seedling resistance with 13 different pathotypes of *P. striiformis*, which were in total virulent against *Yr1, Yr2, Yr3, Yr4, Yr6, Yr7, Yr8, Yr9, Yr17, Yr27, Yr32, YrA, YrSD, YrSO* and *YrSP* (Pathan et al. 2008). This study identified the seedling resistance gene *Yr27* and additional resistance(s) of uncertain identity in Capo and the absence of any seedling resistance gene in Arina. Capo and Arina displayed high levels of resistance at adult plant growth stages in field nurseries, when inoculated with pathotypes virulent to *Yr2*, *Yr3*, *Yr4*, *Yr6*, *Yr7*, *YrSD*, *YrSO*, *YrA* (Pathan et al. 2008).

### Field experiments

#### Leaf rust experiments

233 RILs of population CA and 201 RILs of population CF, the parents and several control lines were tested in different experimental sites over several years. Population CA was evaluated in eight field trials conducted in Austria at Tulln, Probstdorf, Rust, Schmida, and at Fundulea/Romania, Martonvásár/Hungary in 2008 and at Tulln and Rust in 2009. Population CF was tested in four experiments, carried out at Tulln in 2004, 2007 and 2008, and at Probstdorf in 2006. Trials were managed according to local practices of the respective locations. RILs were sown in single or double rows interspersed by a single spreader row. Experiments were artificially inoculated using a local bulk of urediniospores of unidentified pathotypes. Such spore bulks were usually collected in surrounding areas of the experimental sites in the previous season, and, therefore, represent the local pathotype population. Leaf rust epidemics were provoked by spraying or injecting a suspension of urediniospores on or into spreader rows/plants and planting diseased seedlings into spreader rows. The average percentage of leaf area of adult plants covered by leaf rust (leaf rust severity, LrS) was visually estimated according to the scale described by Moll et al. (1996). One or more ratings were done, the latest usually shortly before leaf senescence when the upper leaves were still green and scorable. Statistical analysis was carried out on data from the final disease rating when the disease was usually maximally spread. Information on locations, experimental designs, spreaders, inoculation techniques of individual trials is provided in Online Resource 1.

#### Stripe rust experiments

172 RILs of population CA and 201 RILs of population CF, the parents and several control lines were tested in different experimental sites over several years in altogether five respective six field trials. Stripe rust experiments of population CA were conducted at Tulln/Austria in 2011 and at Atzenbrugg/Austria and Reichersberg/Austria, at Changins/Switzerland and at Cappelle-en-Pévèle/France in 2012. Population CF was evaluated at Reichersberg in 2009 and 2010, at Tulln in 2010 and 2011, and at Atzenbrugg and Changins in 2012. Subsets of 31 RILs of both populations were additionally tested in the United Kingdom at Cambridge and Ickleton in 2012. Agronomic practices followed local standards. Apart from the trial at Cambridge, all experiments were artificially inoculated. Pathotype or pathotype mixtures propagated on seedlings of susceptible genotypes in the greenhouse under controlled conditions were collected and used for inoculation. The highly aggressive Warrior race was already included in experiments Cappelle-en-Pévèle/France in 2012 and United Kingdom at Ickleton in 2012 (see Online Resource 2).  All experiments included a combination of pathotypes, which were, among others, virulent against resistance genes [1, 2, 3, 6, 7, 8, 9, 17, 25, 32], although no single pathotype had this specific virulence combination.  Experiments were inoculated by spraying urediniospore suspensions onto test lines and/or transplanting infected seedlings into the trials. Information on individual experiments, inoculation methods, virulence profiles of applied pathotypes and disease evaluation methods are summarized in Online Resource 2. The average percentage of leaf tissue of adult plant covered by stripe rust (stripe rust severity, YrS) was visually estimated according to the scale described by Moll et al. (1996) when the disease was maximally spread. Differently scored experiments were converted into percentage values.

#### Statistical analysis of field experiments

Analysis of variance (ANOVA) and correlation of field data were calculated in SAS/STAT version 9.2 (SAS Institute Inc 2008). Distribution of residuals was tested for normality using PROC UNIVARIATE applying the Kolmogorov–Smirnov statistic. A Logit transformation was chosen to adjust the stripe rust data to achieve near normality and the statistical analysis of the stripe rust experiments were done on the Logit transformed and the non-transformed datasets. For stripe rust, the presented first order parameters (e.g. means, histograms, additive effects) were obtained from untransformed data, while second order statistics (ANOVAs, broad sense heritability, correlation coefficients, LOD scores and percent explained variance by QTL) were calculated from the transformed data. Analysis of variance (ANOVA) was conducted using the general linear model (GLM) procedure, with all effects fixed. The effects of experiments, as a combination of year and location, replication within experiments, genotype, and genotype-by-experiment interaction were calculated. Broad-sense heritability was estimated from variance components with the equation *H* = σ_G_^2^/(σ_G_^2^ + σ_G×E_^2^/*e* + σ_E_^2^/*en*), where σ_G_^2^ = genotypic variance, σ_G×E_^2^ = genotype-by-experiment interaction variance, σ_E_^2^ = error variance, *e* = number of experiments, and *n* = number of replications (Nyquist 1991). For the estimation of broad-sense heritability, all effects were considered random. Spearman rank-correlation coefficients were estimated for all pair-wise experiment combinations and correlation between stripe rust and leaf rust severity was estimated from means over all experiments.

### Molecular marker analysis and map construction

High-quality genomic DNA was isolated from pooled samples of young leaves from ten plants of each RIL and of the parental lines using the cetyl-trimethyl-ammonium bromide (CTAB) method of Saghai-Maroof et al. (1984). Diversity array technology (DArT) marker assays were performed on parents and on 171 and 178 RILs of populations CA and CF, respectively, by Triticarte Pty. Ltd (Canberra, Australia; http://www.triticarte.com.au). Furthermore, SSR (simple sequence repeat) markers were added primarily at genomic region where QTL were detected or suspected. Six BARC (Song et al. 2005), 36 GWM (Roeder et al. 1998) and 3 WMC (Somers et al. 2004) markers were used in population CA and 7 GWM, 5 BARC and 2 WMC markers in population CF.

Map construction of CA and CF populations was carried out using CarthaGene 1.2-LKH (de Givry et al. 2005). Marker data of both populations were analyzed simultaneously using the command *dsmergor*. This produces consensus data sets sharing marker order, but separate parameter estimates with per dataset distances (CarthaGene user manual). Apparently allelic markers were removed leaving a single representative. Distances between markers in cM were calculated based on the Kosambi mapping function implemented in CarthaGene. Linkage groups were assigned to chromosomes according to information from Triticarte (http://www.triticarte.com.au) and allocated to a specific chromosomal arm by comparing the arrangement of markers with maps published by Marone et al. (2012), Francki et al. (2009), Mantovani et al. (2008), Peleg et al. (2008), Crossa et al. (2007) and Akbari et al. (2006) and maps available in the GrainGenes database (http://wheat.pw.usda.gov/ggpages/maps.shtml).

### QTL analysis

QTL calculations were carried out with R version 3.0.2 (R Development Co re Team 2011) using the R/qtl package 1.28-19–12 (Broman et al. 2003). Missing genotypic information was imputed using the multiple imputation method of Sen and Churchill (2001). Genome wide QTL searches were conducted for each experiment separately and for the overall means across all experiments. Interval mapping was performed using a single QTL genome scan and pairwise epistatic QTL interactions were calculated using a two dimensional QTL scan via Haley–Knott regression (Haley and Knott 1992). LOD significance thresholds of the respective trait and population for type I error rates of *α* < 0.1, *α* < 0.05 and *α* < 0.01 were determined by running 1000 permutations on the single and two dimensional QTL scan. Finally, a multiple QTL mapping (MQM) analysis was performed on the individual environments and on the overall means across environments. Trait and population specific MQM models were fitted including all significant QTL and QTL interactions. The overall fit of the full model against the null model was tested by ANOVA and the estimated additive effect and the percentage of phenotypic variance explained by each QTL were obtained from the MQM analysis. The QTL support interval criterion was determined using a LOD decrease of 1.5 from the maximum LOD position. Linkage groups and LOD bars were drawn with MapChart v2.2 (Voorrips 2002). No QTL analysis was performed on the subsets tested for YrS at Cambridge/GB and at Ickleton/GB. Lines of these sub-populations were grouped according their genotypic information by the number of QTL improving alleles and differences between the means of these groups were compared using the Duncan multiple range test.

## Results

### Trait variation and trait correlations

**Fig. 1:**
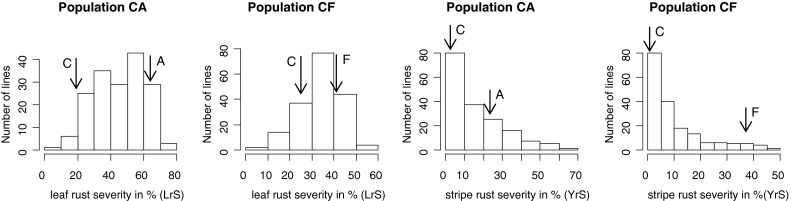
Phenotypic variation in leaf rust and stripe rust severity of the averaged means across all experiments for population Capo × Arina (CA) and Capo × Furore (CF). Frequency distribution of RILs for YrS (%) and LrS (%). Parental scores Capo (C), Arina (A), Furore (F) are indicated by *arrows*

The field studies achieved various levels of infections for both leaf rust and stripe rust. Distribution of lines according their leaf rust and stripe rust severity of the averaged means across all experiments for population CA and CF are depicted in Fig. 1. The phenotypic frequency distribution for YrS differed significantly from normality for both populations (*p* < 0.001) and was skewed towards resistance. The phenotypic frequency distributions for LrS were more continuous (*p* > 0.01) and were slightly skewed towards susceptibility. The population means of the stripe rust experiments were in general lower than those of the leaf rust experiments. The susceptible parents Furore and Arina were consistently more diseased in all leaf rust experiments than the resistant parent Capo. The stripe rust scores for Capo were generally low (0–2.5 YrS), with the exception of the experiment at Cambridge (30 YrS). Infection level of Capo varied from low to medium values (3–47 LrS) in the leaf rust experiments. This is shown in Table 1, where mean values of the parents, means and ranges of the populations and least significant differences of individual experiments are summarized. Spearman rank-correlations between experiments were all significantly different from zero and showed ranges from *r* = 0.58–0.83 and *r* = 0.53–0.89 for the stripe rust experiments and from 0.45–0.78 and 0.39–0.73 for the leaf rust experiments in population CA and CF, respectively (Online Resource 3). Correlations between mean LrS and mean YrS were *r* = 0.58 in population CA and *r* = 0.65 in CF.

**Table 1:** Means of parents, mean, minimum and maximum values of populations, least significant differences at α < 0.05 (LSD) of stripe rust and leaf rust severity

Trait	Site	Country	Year	Parents		Mean	Min	Max	LSD5%
Stripe rust severity (%)									
Population Capo × Furore				Capo	Furore				
Overall mean				0.3	38.5	9.5	0	49	5.6
Reichersberg		AT	2009	0	7.6	2.8	0	18	2.1
Reichersberg		AT	2010	0	17.7	4.7	0	26	2.3
Tulln		AT	2010	0.1	53.0	7.1	0	65	7.4
Tulln		AT	2011	0	45.0	7.9	0	70	6.2
Atzenbrugg		AT	2012	0.75	70.0	20.1	0	75	8.1
Changins		CH	2012	0.75	37.5	16.5	0	55	4.9
Cambridge^a^		GB	2012	30	65.0	43.1	2.5	80	9.3
Ickleton^a^		GB	2012	0	50	15.7	0	63	7.1
Population Capo × Arina				Capo	Arina				
Overall mean				0.1	24.0	16.0	0	65	5.5
Tulln		AT	2011	0	20.0	10.9	0	85	5.2
Atzenbrugg		AT	2012	0	65.0	29.3	0	90	6.9
Reichersberg		AT	2012	0.5	2.0	2.9	0	14	1.5
Cappelle		FR	2012	0	10.0	19.9	0	75	5.2
Changins		CH	2012	0	25.0	15.1	0	73	7.1
Cambridge^a^		GB	2012	30	45.0	33.0	12.5	55	11.5
Ickleton^a^		GB	2012	0	0.1	3.0	0	30	5.4
Leaf rust severity (%)									
Population Capo × Furore				Capo	Furore				
Overall mean				25.5	41.6	34.7	6.5	53	7.5
Tulln		AT	2004	7.0	33.3	22.0	1	45	6.8
Probstdorf		AT	2006	47.5	57.5	53.5	4	80	5.5
Tulln		AT	2007	20.0	28.0	24.2	5	50	10.0
Tulln		AT	2008	27.5	47.5	38.8	4	60	7.2
Population Capo × Arina				Capo	Arina				
Overall mean				18.9	67.7	43.2	4	70	8.7
Martonvásár		HU	2008	20.0	90.0	60.1	0	100	11.6
Fundulea		RO	2008	3.0	90.0	50.7	0	100	-
Schmida		AT	2008	17.3	53.8	31.8	0	60	11.2
Probstdorf		AT	2008	47.5	58.8	51.6	30	65	4.0
Rust		AT	2008	16.9	60.0	45.3	5	70	9.4
Tulln		AT	2008	3.0	26.5	16.7	1	40	5.4
Rust		AT	2009	21.3	82.5	46.7	2	90	11.7
Tulln		AT	2009	21.9	80.0	44.7	3	90	10.5

^a^subset of 31 lines, data not included in overall mean

ANOVA for stripe rust and leaf rust severity resulted in highly significant effects (*p* < 0.0001) for experiment, genotype and genotype × experiment interaction (Online Resource 4). The variation due to the genotypes was high compared to the genotype by experiment variances. This resulted in high heritability values. Broad-sense heritabilities for means over all experiments were *H* = 0.93 for YrS for both populations and were *H* = 0.84 in the CF versus *H* = 0.89 in the CA population for LrS (Tables 2, 3).

**Table 2:** Locations and estimates of QTL for leaf rust and stripe rust severity measured by the percentage of infected leaf area (LrS, YrS) using multiple QTL mapping in population Capo × Arina

QTL^d^	Markers			Stripe rust severity						Leaf rust severity								
			Site	Tulln	Atzen-brugg	Reichers-berg	Cap-pelle	Changins	Mean over all exps	Fun-dulea	Marton-vásár	Probst-dorf	Schmida	Rust	Tulln	Rust	Tulln	Mean over all exps
			Country	AT	AT	AT	FR	CH		RO	HU	AT	AT	AT	AT	AT	AT	
			Year	2011	2012	2012	2012	2012		2006	2008	2008	2008	2008	2008	2009	2009	
*QLr.ifa*-*1B (centromeric)*	1BS	*wPt*-*3103*	LOD	–	–	–	–	–	–	–	8.9	–	–	–	–	–	–	–
	Closest	*Xgwm11*	PV^a^	–	–	–	–	–	–	–	11.4	–	–	–	–	–	–	–
	1BL	*wPt*-*3451*	Add^b^	–	–	–	–	–	–	–	8.9	–	–	–	–	–	–	–
*QYr.ifa*-*2AL QLr.ifa*-*2AL*	Proximal	*wPt*-*8596*	LOD^c^	10.6	16.6	6.1	5.1	4.1	11.9	3.6	11.4	7.6	9.0	5.2	8.9	10.9	13.4	13.6
	Closest	*tPt*-*8937*	PV^a^	14.1	26.8	10.1	7.7	7.2	16.3	5.5	15.2	15.8	17.8	10.2	20.1	22.7	25.7	22.8
	Distal	*Xgwm312*	Add^b^	5.2	13.6	1.0	6.6	4.3	6.0	5.5	10.2	2.5	6.4	5.1	5.4	12.0	12.9	7.4
*QLr.ifa*-*2BS*	Proximal	*Xgwm120*	LOD^c^	–	–	–	–	–	–	5.6	13.9	3.7	4.1	8.1	4.3	6.1	4.3	8.8
	Closest	*wPt*-*6471*	PV^a^	–	–	–	–	–	–	8.3	19.2	7.2	7.6	16.4	9.1	11.7	7.2	13.8
	Distal	*wPt*-*6932*	Add^b^	–	–	–	–	–	–	6.7	11.6	1.6	4.0	6.0	3.4	8.2	6.5	5.7
*QYr.ifa*-*2BL*	Proximal	*wPt*-*7360*	LOD^c^	12.8	10.1	11.0	8.3	5.6	12.2	–	–	–	–	–	–	–	–	–
	Closest	*wPt*-*733641*	PV^a^	17.5	14.8	19.6	13.1	10.1	16.8	–	–	–	–	–	–	–	–	–
	Distal	*wPt*-*733641*	Add^b^	6.5	10.7	1.4	7.5	4.0	6.1	–	–	–	–	–	–	–	–	–
*QLr.ifa*-*2DS*	Proximal	*Xwmc25.2*	LOD^c^	–	–	–	–	–	–	15.7	–	–	–	–	–	–	–	–
	Closest	*wPt*-*6780*	PV^a^	–	–	–	–	–	–	28.5	–	–	–	–	–	–	–	–
	Distal	*wPt*-*666162*	Add^b^	–	–	–	–	–	–	11.8	–	–	–	–	–	–	–	–
*QYr.ifa*-*3AS*	Proximal	*wPt*-*0714*	LOD^c^	6.3	–	3.4	4.5	–	4.5	–	–	–	–	–	–	–	–	–
	Closest	*wPt*-*7890*	PV^a^	7.8	–	5.4	6.7	–	5.6	–	–	–	–	–	–	–	–	–
	Distal	*wPt*-*9634*	Add^b^	−5.2		−0.8	- 6.9	–	−4.0	–	–	–	–	–	–	–	–	–
*QYr.ifa*-*3BS* *QLr.ifa*-*3BS*	Proximal	*Xbarc133*	LOD^c^	8.8	5.1	*2.5*	8.8	7.9	9.2	3.4	4.4	–	*2.3*	3.0	–	–	*2.5*	4.6
	Closest	*wPt*-*10192*	PV^a^	11.3	7.0	*3.9*	13.9	14.7	12.1	5.2	5.2	–	*4.1*	5.7	–	–	*4.1*	6.8
	Distal	*Xgwm389*	Add^b^	4.1	6.1	*0.5*	6.7	5.5	4.5	4.9	5.8	–	*2.9*	3.6	–	–	*5.3*	4.0
*QLr.ifa*-*5BL*	Proximal	*wPt*-*7006*	LOD^c^	–	–	–	–	–	–	–	3.7	–	3.8	–	–	3.1	3.6	5.0
	Closest	*wPt*-*6971*	PV^a^	–	–	–	–	–	–	–	4.5	–	7.0	–	–	5.7	6.0	7.4
	Distal	*wPt*-*0295*	Add^b^	–	–	–	–	–	–	–	−5.4	–	−3.9	–	–	−5.8	−6.4	−4.4
LOD of the full model			LOD	31.8	27.6	21.2	24.2	17.8	31.3	22.9	32.2	9.4	15.8	15.1	11.2	16.1	19.9	24.7
Total variance explained (%)			PV^a^	57.5	52.4	43.5	47.9	38.2	56.9	48.2	58.4	22.4	34.7	34.0	26.0	36.1	41.8	48.6
Heritability/repeatability			*H*	0.95^g^	0.94^g^	0.83^g^	0.90^g^	0.78^g^	0.93^f^	–^h^	0.86^g^	0.68^g^	0.60^g^	0.67^g^	0.79^g^	0.89^g^	0.87^g^	0.89^f^

^a^Significance codes P: 0 < ‘standard’ < 0.001 < *‘italic’* < 0.01; non-significant data are not presented

^b^Percentage of phenotypic variance explained by the QTL

^c^Positive additive effects denote LrS/YrS-reducing effect of the Capo allele; QTL effect was estimated as the difference in the mean between the two homozygous QTL genotypes of the untransformed data

^d^QTL name described by rust disease and chromosome or chromosome arm

^f^Broad-sense heritability

^g^Repeatability

^h^Not estimated

**Table 3:** Locations and estimates of QTL for leaf rust and stripe rust severity measured by the percentage of infected leaf area (LrS, YrS) using multiple QTL mapping in population Capo × Furore

^d^QTLand QTL:QTL interaction	Markers		Stripe rust severity								Leaf rust severity				
			Site	Reichers-berg	Reichers-berg	Tulln	Tulln	Atzen-brugg	Changins	Mean over all exps	Tulln	Probst-dorf	Tulln	Tulln	Mean over all exps
			Country	AT	AT	AT	AT	AT	CH		AT	AT	AT	AT	
			Year	2009	2010	2010	2011	2012	2012		2004	2006	2007	2008	
*QYr.ifa*-*2BL QLr.ifa*-*2BL*	Proximal	*Xwmc317*	LOD^a^	31.1	37.0	24.7	29.0	27.1	35.0	45.4	5.4	5.7	–	5.8	6.8
	Closest	*wPt*-*6643*	PV %^b^	47.7	58.0	37.0	40.2	39.4	43.8	54.5	8.8	8.7	–	11.0	10.5
	Distal	*wPt*-*2425*	Add^c^	2.3	4.4	6.9^e^	7.6^e^	14.9	10.3	7.7	3.3	3.9	–	4.2	3.2
*QYr.ifa*-*3BS QLr.ifa*-*3BS*	Proximal	*Xbarc133*	LOD^a^	13.8	10.2	24.6	27.2	6.3	29.7	27.8	17.6	21.2	4.2	11.8	20.2
	Closest	*wPt*-*10192*	PV %^b^	16.6	10.9	36.8	36.8	6.8	34.5	25.8	34.2	40.1	10.3	24.3	37.6
	Distal	*Xgwm389*	Add^c^	1.1	1.6	7.9^e^	8.3^e^	4.4	7.5	4.6	6.4	8.2	3.2	6.1	5.9
*QYr.ifa*-*5A*	Proximal	barc117	LOD^a^	2.7	–	–	5.3	16.1	*2.0*	10.3	–	–	–	–	–
	Closest	*wPt*-*3509*	PV %^b^	2.8	–	–	5.3	20.0	*1.6*	7.4	–	–	–	–	–
	Distal	*wPt*-*2426*	Add^c^	0.8	–	–	3.3	10.4	*1.9*	3.1	–	–	–	–	–
*QYr.ifa*-*2BL* *QYr.ifa*-*3BS*	wPt-6643:wPt-10192		LOD^a^	–	–	9.8	9.3	–	–	–	–	–	–	–	–
			PV %^b^	–	–	11.9	9.8	–	–	–	–	–	–	–	–
LOD of the full model			LOD	36.8	39.4	34.1	39.5	36.6	46.8	54.5	20.4	23.3	4.2	14.9	23.0
Total variance explained (%)			PV %^b^	61.4	63.9	58.6	64.0	61.2	70.2	75.6	41.2	45.3	10.3	32.0	45.3
Heritability/repeatability			*H*	0.77^g^	0.87^g^	0.80^g^	0.89^g^	0.89^g^	0.87^g^	0.93^f^	0.72^g^	0.88^g^	0.34^g^	0.77^g^	0.84^f^

^a^Significance codes *P*: 0 < ‘standard’ < 0.001 < *‘italic’* < 0.01; non-significant data are not presented

^b^Percentage of phenotypic variance explained by the QTL

^c^Positive additive effects denote LrS/YrS-reducing effect of the Capo allele; QTL effect was estimated as the difference in the mean between the two homozygous QTL genotypes of the untransformed data

^d^QTL name described by rust disease and chromosome or chromosome arm

^e^Estimated single QTL effect and QTL by QTL interaction effect not unambiguously distinguishable

^f^Broad-sense heritability

^g^Repeatability

### Linkage maps

DArT and SSR markers generated a total of 674 and 710 polymorphic markers in population CA and CF, respectively. The high number of redundant DArT markers reduced the final number of unique marker loci to 432 in the CA map and to 310 in the CF map, of which 129 markers were present in both populations. The total map length of population CA was 1,644 cM, resulting in an average distance of 3.8 cM. Markers fell into 34 linkage groups covering 635 cM on genome A, 727 cM on genome B and 256 cM on genome D. Twenty-six cM could not be unambiguously assigned to a chromosome, no linkage group could be attributed to chromosome 4D. Capo and Furore are related cultivars, which is reflected in the relatively shorter total map length of 859 cM, with a 2.8 cM average marker distance for CF population. Markers of population CF fell into 31 linkage groups covering 230 cM on the A genome, 537 cM on the B genome, 48 cM on the D genome, whilst 44 cM remained unassigned. All chromosomes were represented, but chromosomes on the D genome had lower marker coverage.

### QTL analysis

**Fig. 2:**
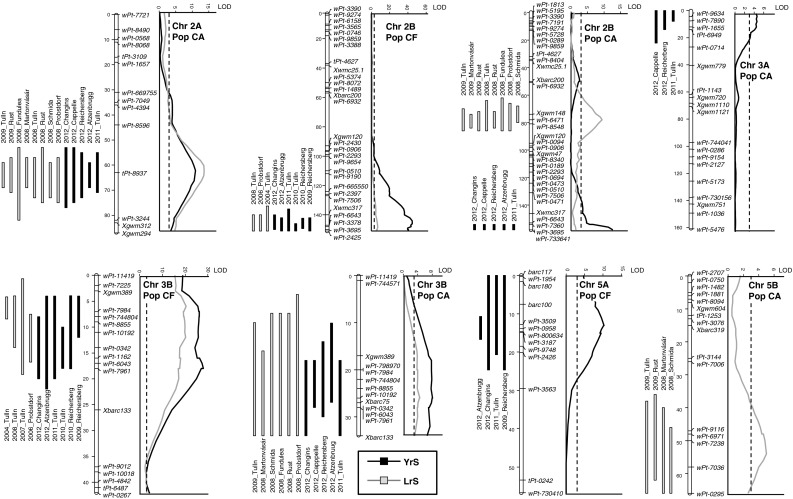
Maps of linkage groups harbouring QTL identified in multiple experiments. QTL for stipe rust severity (YrS) and leaf rust severity (LrS) are determined by the MQM model. LOD profiles obtained from the averaged mean of all experiments are given on the right. *Bars* of the QTL support interval for the respective experiments are on the left. *Bar size* indicates a LOD decrease of 1.5 from maximum LOD. The *dashed lines* represent the LOD 3 value

**Fig. 3:**
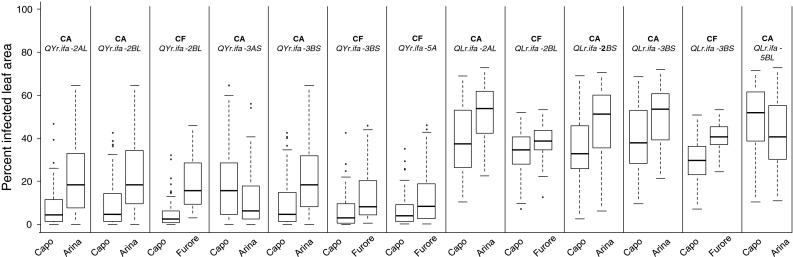
*Boxplot* of QTL effects for QTL identified in multiple experiments. Genotypes were classified by allele status of the closest markers to the corresponding QTL. Percentage of infected leaf area of stripe rust (YrS) and leaf rust (LrS) are based on average mean values across experiments. Medians are indicated by *solid lines*

For stripe rust experiments the analysis was run with both the transformed and non-transformed datasets. Basically the same results were obtained with only small differences in the calculated estimates of detected QTL. The results presented here refer to the transformed dataset. All QTL identified, their positions and statistical parameters for population CA and CF are summarized in Table 2 and Table 3, respectively. QTL identified in multiple experiments are depicted in Fig. 2 and boxplots of the effect of contrasting alleles at these QTL are illustrated in Fig. 3. LOD profiles of QTL are based on overall means, size of the QTL support intervals of the individual experiments are given. For graphical simplicity no co-segregating markers are shown in Fig. 2. More detailed information on these linkage groups, including all co-segregating markers, their positions and genetic distances are provided in Online Resource 5.

### QTL for resistance to stripe rust

**Fig. 4:**
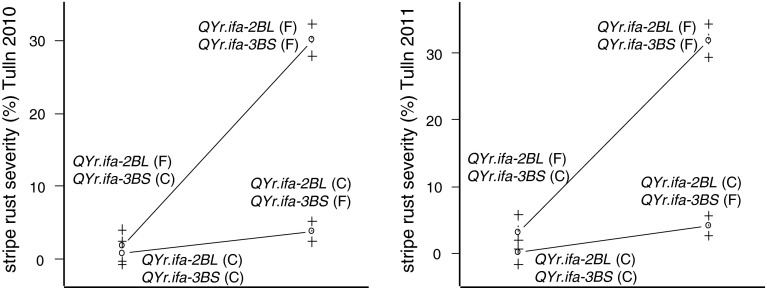
Two-way interaction plots between *QYr.ifa*-*2BL* and *QYr.ifa*-*3BS* for each genotypic allele combination of Capo (C) and Furore (F). The phenotypic means are plotted, with *error bars* at ±1 SE

Five versus six field experiments were conducted to evaluate YrS in population CA and CF, respectively. Five genomic regions on five chromosomes—2AL, 2BL, 3AS, 3BS, 5A, designated as *QYr.ifa*-*2AL, QYr.ifa*-*2BL, QYr.ifa*-*3AS, QYr.ifa*-*3BS, QYr.ifa*-*5A*—were associated with YrS (Tables 2, 3; Fig. 2). Capo alleles conferred resistances at *QYr.ifa*-*2AL, QYr.ifa*-*2BL, QYr.ifa*-*3BS* and *QYr.ifa*-*5A*, and the Arina allele at *QYr.ifa*-*3AS* (Fig. 3). Of these, *QYr.ifa*-*2BL* and *QYr.ifa*-*3BS* were identified in both populations, *QYr.ifa*-*2AL* and *QYr.ifa*-*3AS* in population CA and *QYr.ifa*-*5A* in population CF only. Across experiments, by far the greatest effect was contributed by the QTL on 2BL. *QYr.ifa*-*2BL* explained between 37 and 58 % of the total phenotypic variance in population CF (Table 3). Its effect in population CA was less pronounced but still high, contributing 10–20 % to the explained variability (Table 2). *QYr.ifa*-*2BL* is located on the distal end of the long arm and achieved the highest estimates on overlapping intervals at marker *wPt*-*6643* in CF and at marker *wPt*-*733641* in the CA population. QTL support interval on 3BS spanned over 15 cM and was flanked by *Xgwm389* and *Xbarc133* in both populations. *QYr.ifa*-*3BS* had a major effect in CF (Table 3), and a moderate effect in population CA (Table 2). *QYr.ifa*-*2AL* was located within a 20 cM interval, with *tPt*-*8937* as peak marker. It was an important source of resistance in all experiments of population CA accounting for 7 up to 27 % of the observed variation (Table 2). The corresponding interval on 2AL was not segregating and thus not observable in population CF. A QTL on chromosome 5A contributed to stripe rust development in four of six experiments in population CF only. The confidence interval spans a distance of 20 cM and is flanked by *Xbarc117* on the short arm and *wPt*-*2426* on the long arm of chromosome 5A. The influence of *QYr.ifa*-*5A* was more variable, with a notable strong effect in one experiment (20 % PV), but had rather small effects in the other experiments (1.6–5 PV %) (Table 3). A minor QTL, with resistance contributed by the Arina allele, was identified on the short arm of chromosome 3A in three of the five experiments of population CA (Table 2). QTL predominantly acted in an additive manner, although significant epistatic QTL interactions were found in two experiments of population CF (Tulln 2010, Tulln 2011) between *QYr.ifa*-*2BL* and *QYr.ifa*-*3BS*. Either of these QTL alone substantially improved resistance (Table 3; Fig. 4). When occurring together, resistance improved slightly relative to the single QTL effect in cases where both favorable alleles were combined, but decreased heavily when both were absent (Fig. 4). Significant interaction between *QYr.ifa*-*2BL* and *QYr.ifa*-*3BS* were additionally found in experiment Reicherberg 2010 and Changings 2010 when using the untransformed dataset (results not shown). No QTL by QTL interactions were found in population CA.

QTL information, particularly allele status at *QYr.ifa*-*2BL* and *QYr.ifa*-*3BS* for populations CF, and allele status of at *QYr.ifa*-*2AL*, *QYr.ifa*-*2BL*, *QYr.ifa*-*3AL* and *QYr.ifa*-*3BS* for population CA, were used to study the effects of stripe rust QTL in the United Kingdom, an environment with known high pressure of stripe rust (Online Resource 6). Results of population CF clearly separated groups of lines with none, one or two stripe rust resistance QTL alleles, reaching 56, 29, and 14 % YrS, respectively. More QTL were involved in resistance in population CA. Here lines with no QTL were significantly more diseased than all other QTL groups. Lines harboring one or two resistance improving QTL alleles were more resistant than lines with no positive allele. The group of lines with four, three or two positive alleles was significantly less infected than lines with one or no stripe rust resistance QTL.

### QTL for resistance to leaf rust

LrS was tested in eight respective four experiments of population CA and CF. QTL analysis resulted in five QTL assigned to chromosome arms 2AL, 2BL, 2BS, 3BS and 5BL (*QLr.ifa*-*2AL*, *QLr.ifa*-*2BL*, *QLr.ifa*-*2BS*, *QLr.ifa*-*3BS*, *QLr.ifa*-*5BL*), which were found in multiple experiments (Tables 2, 3; Figs. 2, 3). Besides these repeatedly detected QTL, two further QTL, allocated to chromosomes 1B and 2DS (*QLr.ifa*-*1B, QLr.ifa*-*2DS*) were identified in single experiments only. QTL profiles of *QLr.ifa*-*1B* and *QLr.ifa*-*2DS* and boxplots illustrating effects of contrasting alleles are presented in Online Resource 7. Most notably, *QLr.ifa*-*2AL*, *QLr.ifa*-*2BL* and *QLr.ifa*-*3BS* coincided with QTL for YrS. *QLr.ifa*-*3BS* was found in both populations on matching intervals, whereas *QLr.ifa*-*1B*, *QLr.ifa*-*2AL*, *QLr.ifa*-*2BS*, *QLr.ifa*-*2DS* and *QLr.ifa*-*5BL* appeared only in population CA, and QTL on *QLr.ifa*-*2BL* was unique for population CF. The Capo allele improved resistance except for *QLr.ifa*-*5BL*. Leaf rust resistance in the population CF was predominantly controlled by the QTL on 3BS which explained 10–34 % of the phenotypic variance (Table 3). *QLr.ifa*-*3BS* was less effective in population CA and accounted for 4–6 PV % in five out of eight experiments (Table 2). *QLr.ifa*-*2BL* was revealed in three of the four CF experiments and showed moderate effects. This QTL coincided with *QYr.ifa*-*2BL*, a major source of resistance in the stripe rust experiments (Table 3). *QLr.ifa*-*2AL* and *QLr.ifa*-*2BS* were consistently identified in experiments of all years and locations in the population CA and explained on average 23 and 14 % of the phenotypic variance, respectively (Table 2). *QLr.ifa*-*2BS* spanned a 15 cM interval and reached maximum values at marker *wPt*-*647*. The corresponding genomic region of QTL of *QLr.ifa*-*2BS* was not polymorphic over a distance of 30 cM in population CF (Fig. 2, Online Resource 5). A minor effect QTL on the distal end of chromosome 5BL appeared only in population CA and was detected in four of the eight experiments. In this case, the susceptible parent Arina conferred the resistance allele (Table 2). Environment specific QTL were identified in the centromeric region of chromosome 1B with *Xgwm11* as peak marker and on the short arm of 2D distal *to Xwmc25.2*. *QLr.ifa*-*2DS* was a major source of resistance in the experiment Fundulea in 2008 and explained 28 % of the phenotypic variance. *QLr.ifa*-*1B* contributed 11 % of the explained variability in the experiment Martonvásár in 2008 (Table 2, Online Resource 7).

When comparing the results of the two populations the following can be summarized: Capo allele contributed the resistance allele for most QTL, Arina for two QTL, while Furore did not contribute any resistance allele. Three QTL, namely *QYr.ifa*-*2BL*, *QYr.ifa*-*3BS* and *QLr.ifa*-*3BS* were identified in both populations. Two QTL with resistance conferred by the Capo allele were identified in population CF only (*QLr.ifa*-*2BL*, *QYr.ifa*-*5A*). Seven QTL were identified in population CA only. In these cases either the genomic intervals were not polymorphic in population CF (*QYr.ifa*-*2AL*, *QLr.ifa*-*2AL*, *QLr.ifa*-*2BS*), population CF was not tested at the specific environments (*QLr.ifa*-*.2DS*, *QLr.ifa*-*1B*), or the resistance was conferred by the Arina allele (*QLr.ifa*-*5BL*, *QYr.ifa*-*3AS*).

## Discussion

Capo combines high resistance to stripe rust with moderate resistance to leaf rust and has maintained good resistance levels since its release in 1989 despite extensive cultivation. The reportedly high level of resistance to stripe rust and moderate level to leaf rust was validated in the present study. The key growing area of Capo is conducive to leaf rust, whereas stripe rust occurs only sporadically. Leaf rust symptoms did not show an immune reaction, but a typical quantitative type of resistance. In contrast to Capo’s long lasting partial resistance to leaf rust, it remains to be shown whether the exhibited stripe rust resistance will persist over time. So far, Capo sustains its high resistance to stripe rust even under the severe stripe rust epidemic in spring 2014, with the aggressive Warrior race as the dominant pathotype across Europe. Previous multi-pathotype screenings provided evidence, that Capo carries uncharacterized resistances for leaf rust and stripe rust in addition to the resistance genes *Lr13* and *Yr27* (Winzeler et al. 2000; Pathan and Park 2006; Pathan et al. 2008). Accordingly, our study confirmed the presence of several QTL for resistance to leaf rust and stripe rust in Capo. Frequency distribution of RILs for leaf rust severity appeared continuous, suggesting a polygenic and complex genetic control. Indeed, altogether seven different chromosomal regions were associated with LrS, although not all QTL were consistently identified across populations and environments. It is likely that the durable resistance to leaf rust arises from a combination of several low to moderate effect QTL. The rust pathogen can easily adapt its genotype by mutation when facing a single-gene resistance, but is much less likely to overcome several resistance genes in combination (Hovmøller 2001). Frequency distribution of RILs for YrS was continuous but skewed towards low infection and was regulated by five different QTL. The highly significant phenotypic correlations between LrS and YrS in both populations suggest that resistances to these diseases probably are under some common genetic control. As expected, several coinciding QTL for leaf rust and stripe rust resistance were found. Subpopulations of CA and CF were tested in the United Kingdom to verify if the identified QTL are effective in an environment highly conducive to stripe rust epidemics. The results confirmed that the detected stripe rust resistance QTL confers quantitative resistance also on the United Kingdom.

### Multiple-disease-resistance QTL effective for leaf rust and stripe rust

Genotypes possessing multiple-disease-resistance QTL, either closely linked in coupling phase or the pleiotropic effect of a single gene, are particularly valuable in breeding, as these resistances will be inherited simultaneously. QTL mapping identified genomic regions on chromosomes 2AL, 2BL and 3BS which were associated with both LrS and YrS and it was always the Capo allele which improved resistance. Wheat homoeologous group 2 chromosomes are a rich source of resistance to both, stripe rust and leaf rust. From the currently designated *Lr* and *Yr* genes 18 and 12, respectively have been mapped to group 2 (McIntosh et al. 2012). Homoeologous group 2 was an important source of resistance in the analyzed mapping populations as well, since QTL were detected on all group 2 chromosomes. QTL for LrS and YrS co-located to identical intervals on 2AL and were consistently observed in all experiments. Due to lack of polymorphism in population CF at the respective QTL interval, this QTL was only identified in population CA. Resistance genes *Yr1* (Macer 1966) and *Yr32* (Eriksen et al. 2004) are assigned to chromosome 2AL. Paillard et al. (2012) reported a minor QTL, which marginally overlapped with the QTL interval identified in our study. Resistance was derived from the cultivar Taldor; and there is evidence, that this QTL is race-specific as it was efficient against one single pathotype only. The experiments of the present study were inoculated with different pathotypes or mixtures thereof (Online Resource 2) and the identified resistance QTL was effective against all applied *Yr* pathotypes. Capo is susceptible to pathotypes virulent against *Yr1* and *Yr32* (Pathan et al. 2008), thus the detected QTL may be novel. *Lr38*, a resistance derived from *Thinopyrum intermedium* (McCallum et al. 2012) and *LrTt1,* resistances derived from *T. timopheevii* (Leonova et al. 2010) have been up to now the only leaf rust resistance genes reported on the long arm of the 2A chromosome. The resistance donor Capo does not contain these translocations, therefore the 2A QTL identified in the current study is likely to be a novel resistance locus for both stripe and leaf rust.

By far the largest contribution to YrS was obtained from *QYr.ifa*-*2BL*. It was detected in all experiments of population CA and CF and had a remarkably strong effect in population CF (Tables 2, 3; Fig. 2). Besides its strong contribution to stripe rust resistance, a minor effect for LrS co-located in population CF (Table 3; Fig. 2). Markers linked with resistance mapped on the long arm of the 2B chromosome 10 cM distal to *Xgwm317*. Ordering of DArT markers involved in resistance is in agreement with maps of Mantovani et al. (2008); Francki et al. (2009); Crossa et al. (2007) placing the markers to the distal end of chromosome 2BL, but the relevant markers are more proximal in the consensus map of Marone et al. (2012). Association analysis of CIMMYT elite spring wheat revealed association for both, stripe rust and leaf rust, with DArT markers *wPt*-*3378* and *wPt*-*7360* (Crossa et al. 2007). These markers are located within the confidence interval of *QYr.ifa*-*2BL*/*QLr.ifa*-*2BL*. While there is up to now no designated *Yr* gene reported at the distal end of 2BL, there are two *Lr* genes, *Lr50* and *Lr58*, both derived from alien sources, assigned to the terminal end of 2BL. *Lr50* is a resistance transferred to wheat from *T. timopheevii* subsp. *armeniacum* (Brown-Guedira et al. 2003) and *Lr58* is derived from *Aegilops triuncialis* (Kuraparthy et al. 2007). Neither of these resistances had been deployed in any cultivar when Capo was developed (Brown-Guedira et al. 2003).

QTL for stripe rust and leaf rust co-located on chromosome 3BS and mapped between *Xgwm389* and *Xbarc133*. This interval was consistently associated with YrS in all experiments of both populations and consistent for LrS in population CF, but it was less effective to LrS in population CA. This genomic region influences development of many different fungal diseases and has been repeatedly reported in mapping studies of stripe rust, leaf rust, stem rust, powdery mildew and Fusarium head blight. For example, leaf rust gene *Lr27* (Nelson et al. 1997), stem rust gene *Sr2* (Spielmeyer et al. 2003; Kota et al. 2006), stripe rust gene *Yr30* (Singh et al. 2001), *YrRub* (Bansal et al. 2010), powdery mildew (Mago et al. 2011) and Fusarium head blight gene *Fhb1* (Liu et al. 2008) are located in this interval. Tight linkage exists between partial APR gene *Sr2*, seedling resistance gene *Lr27*, partial APR gene *Yr30*, pseudo-black chaff (PBC) (Singh and McIntosh 1984) and powdery mildew (Mago et al. 2011). PBC causes a genotype-dependent pigmentation of stems and/or glumes and has long been used as a phenotypic marker for *Sr2* (McFadden^1939^; Hare and McIntosh 1979). Mago et al. (2011) hypothesize, that a single gene on chromosome arm 3BS may be responsible for resistance to these three fungal pathogens. Capo, the resistance donor on 3BS, does neither contain leaf rust gene *Lr27* (Park et al. 2001) nor stem rust gene *Sr2* (Pathan 2007) and does not develop PBC. Hence *QLr.ifa*-*3BS* and *QYr.ifa*-*3BS* identified in our study appear to be distinct to the above multiple-disease-resistance genes. The resistance gene *YrRub,* which is likely identical to *Yr4*, was mapped on 3BS close to *Xgwm75* (Bansal et al. 2010). Multi-pathotype screening for stripe rust did not detect *Yr4* in Capo (Pathan et al. 2008). Furthermore, all pathotype populations used for inoculation were virulent to *Yr4* (Online Resource 2). The identified *QYr.ifa*-*3BS* therefore differs from *Yr4*. Stripe rust QTL have been repeatedly identified in this region (Boerner et al. 2000; Singh et al. 2000b; 2001; Suenaga et al. 2003; William et al. 2006; Khlestkina et al.^2007^; Dedryver et al. 2009; Lowe et al. 2011b; Hao et al. 2011). Ingala et al. (2012) identified gene *LrSV2,* which coincides with *Lr27*, but unlike the seedling gene *Lr27*, *LrSV2* is effective at adult plant stage only. Additional studies are necessary to precisely locate the QTL on 3BS, to elucidate the relationship to other resistance genes and to investigate if *QLr.ifa*-*3BS* and *QYr.ifa*-*3BS* are closely linked or a single pleiotropic gene. Potentially, all three identified multiple-disease-resistance QTL contribute novel resistance sources for stripe rust and leaf rust.

Studies for epistatic interaction provide additional information on desirable and undesirable combinations of genes. The present study revealed epistatic additive x additive interaction for *QYr.ifa*-*2BL* and *QYr.ifa*-*3BS* in two experiments of population CF. In this particular case, the presence of one favorable allele neutralized much of the effect of the other favorable allele. Similarly, Yang et al. (2013) observed, that combinations of specific *Yr* genes, when occurring together, had the same disease severity as lines containing either of the loci alone and Lowe et al. (2011b) found, that individual effects of QTL, when combined, were greater in the absence of resistant alleles from the other. In wheat lines with combinations of major resistance genes, usually the gene with greatest resistant infection type is epistatic to genes with less resistant infection types (Bolton 2008). However, our results indicate that pyramiding of these QTL will improve overall resistance. The effect of *QYr.ifa*-*2BL* varied from moderate to major, whereas effect of *QYr.ifa*-*3BS* was minor to moderate. This suggests that *QYr.ifa*-*2BL* is a major and putatively race-specific resistance gene, while *QYr.ifa*-*3BS* may or may not be race-specific. The identification of a major gene for YrS verified the finding of Pathan et al. (2008), that Capo carries an uncharacterized seedling resistance gene in addition to *Yr27*.

### QTL effective for resistance to leaf rust only

The *QLr.ifa*-*2BS* was found in population CA only. It mapped close to the centromere near *Xgwm148* (Fig. 2). The corresponding chromosomal region was not polymorphic in population CF, accordingly no QTL was detected. *Lr13, Lr16, Lr23*, *Lr35*, *Lr48* all appear to be close to *Xgwm148* (Maccaferri et al. 2010) near to the centromere and overlapping with *QLr.ifa*-*2BS*. *Lr13* (Bansal et al. 2008)*, Lr16* (McCartney et al. 2005), *Lr23* (Nelson et al.^1997^), *Lr35* (Seyfarth et al. 1999) are located on 2BS, while *Lr48* has been reported on both the short arm (Bansal et al. 2008) and on the long arm of chromosome 2B (Singh et al. 2011b). *Lr13* has been one of the most widely distributed resistance genes worldwide (McIntosh et al. 1995); in contrast, *Lr35* had not been used in modern cultivars when Capo was released. A screening survey of European winter wheat germplasm for *Lr* genes confirmed the presence of *Lr13* and the absence of *Lr16* and *Lr23* in both, Capo and Arina (Winzeler et al. 2000; Park et al. 2001). Presumably Furore also carries *Lr13*, because the corresponding genomic region was monomorphic between Capo and Furore. It is therefore likely that both mapping populations are fixed for *Lr13*. In addition virulence to *Lr13* appears widespread in Europe (Mesterhazy et al. 2000) and was with high probability present in our leaf rust populations. Whether or not *Lr13* had a modulating effect on the leaf rust resistance QTL detected in this study remains unknown. Several QTL studies (Messmer et al. 2000; Xu et al. 2005a, b; Leonova et al. 2007; Rosewarne et al. 2008; Prins et al. 2011) detected QTL of various leaf rust related traits at a similar location. This repeated finding highlights the importance of the centromeric region of 2BS for leaf rust resistance. Whether or not *QLr.ifa*-*2BS* corresponds to any of these previously reported genes or QTL is not yet clarified. In the current study, a QTL for LrS near the telomere of 2DS was detected in the experiment conducted in Romania only. Interestingly, while all other QTL identified in this study were also significant, the 2DS QTL had the most enhanced effect in this particular environment. Mesterhazy et al. (2000) conducted a virulence survey of the wheat leaf rust pathogen in Europe and verified a great diversity in the European population of the wheat leaf rust fungus. As individual leaf rust experiments were inoculated with a mixture of pathotypes collected in surrounding areas of the experimental sites, the used pathogen populations may differ in their virulence profiles. This would suggest that Capo has a race specific response at this interval. Several leaf rust genes are assigned to 2DS of which *Lr15*, *Lr22* (alleles *Lr22a, Lr22b)* and *Lr39* have been mapped to telomeric regions of chromosome 2DS (Dholakia et al. 2013; Hiebert et al. 2007; Raupp et al. 2001), whereas *Lr2* (alleles *Lr2a*, *Lr*2*b*, *Lr*2*c*) is more proximal (McIntosh et al. 1995). Capo does not carry *Lr2a*, *Lr2c* and *Lr15* (Winzeler et al. 2000; Park et al. 2001). *Lr39* and *Lr22a* were introgressed from *T. tauschii* (Raupp et al. 2001; Rowland and Kerber 1974) and *Lr15* and *Lr22b* originate from *T. aestivum* and were first described in Kenya W1483 (Luig and McIntosh 1968) and in the old Canadian cultivar Marquis (Bartos et al. 1969), respectively. Considering the pedigree of Capo, it is unlikely that the detected QTL is equivalent to *Lr22a* or *Lr39* but the pedigree of Capo includes Marquis (Martynov et al. 2006), therefore Capo potentially contains *Lr22b*. Further studies are necessary to clarify whether or not the resistance QTL on 2DS corresponds to *Lr22b*. *QLr.ifa*-*1B* was only observed in the experiment conducted in Hungary. The flanking markers *wPt*-*3103* and *wPt*-*3451* are located on the short and long arm of chromosome 1B respectively (Marone et al. 2012) placing the QTL near the centromere. Currently, there are seven leaf rust resistance genes and two leaf rust QTL assigned to chromosome 1B (McIntosh et al.^2012^), of which *Lr33* (Dyck et al. 1987), *Lr44 (*Dyck and Sykes 1994), *Lr71* (Singh et al. 2013), *LrZH84* (Zhao et al. 2008), *QLr.ifa*-*pser.1BL* (Li and Bai 2009) and *QLr.ifa*-*sfr*-*1B* (Messmer et al. 2000) are all mapped close to the centromere. Unfortunately, with the present study we cannot conclude if *QLr.ifa*-*1B* corresponds to any of these genes or QTL. Although *QLr.ifa*-*2DS* and *QLr.ifa*-*1B* can be useful in certain environments, these QTL should be deployed in combination with other genes, as they may be of little use in regions where the corresponding virulence is present. *QLr.ifa*-*5BL* contributed to leaf rust resistance in two of five experiments in population CA. Markers associated with leaf rust mapped to the distal end of the long arm of chromosome 5B according maps of Marone et al. (2012) and Crossa et al. (2007). So far no leaf rust gene was reported in this interval. However, association studies of wheat landraces identified marker-leaf rust associations at this genomic region (Bansal et al. 2013).

### QTL effective for resistance to stripe rust only

Pathan et al. (2008) evaluated stripe rust resistance of European wheat cultivars and reported high adult plant resistance for Arina, while in our trials Arina was moderately susceptible. *QYr.ifa*-*3AS* was the only QTL with an Arina derived resistance improvement. It had a minor effect and was significant in three of five experiments. Associations between chromosome 3A and stripe rust have been reported in a few studies, although no stripe rust gene has been assigned to 3A so far. Lillemo et al. (2008) and Roswarne et al. (2012) identified minor effect QTL, derived from cv. Saar and cv. Avocet, which coincide with *QYr.ifa*-*3AS* in the present study and an association analysis of CIMMYT elite spring wheat germplasm identified several markers associated with stripe rust within the support interval of *QYr.ifa*-*3AS* (Crossa et al. 2007). Some common ancestors are involved in the pedigree of Arina compared to the resistance donor Saar and Avocet (http://genbank.vurv.cz/wheat/pedigree), thus these cultivars potentially share a common resistance allele. A QTL on chromosome 5A influenced stripe rust response in five of eight experiments in population CF. *QYr.ifa*-*5A* mapped in the centromeric region of 5A. It was not possible to unambiguously assign *QYr.ifa*-*5A* to a chromosomal arm, but the QTL clearly resides close to the centromere. Fang et al. (2011) reported a QTL on the centromeric region of the long arm derived from cultivar Jagger and Quan et al. (2013) identified a QTL on the centromeric region of the short arm of chromosome 5A derived from a Chinese wheat land race. Both QTL are located within the confidence interval of *QYr.ifa*-*5A*.

## Conclusions

Breeding for rust resistance is a continuous effort. New races regularly overcome race-specific resistance genes and novel resistances need to be integrated into breeding germplasm to maintain a required resistance level. The present study identified seven QTL for leaf rust and five QTL for stripe rust resistance and confirmed Capo as an important source for improving rust resistance. The long-lasting resistance of Capo apparently rests upon a combination of several minor and major genes. This finding confirms that the genetic architecture of durable resistant cultivars is complex, usually modulated by a set of genes rather than by a single major gene. QTL on 2AL, 2BL and 3BS were associated with both, leaf rust and stripe rust, either through close linkage or pleiotropy. Deploying such multiple-disease resistances in breeding is particularly advantageous, as they enable improvement of stripe rust and leaf rust resistance simultaneously. The study identified several resistance alleles, but further genetic tests are required to prove, whether the detected QTL correspond to already published genes. But regardless of whether they refer to any previously reported gene or QTL, they are a valuable resistance source embedded in a high quality and successfully deployed cultivar ready for use as a parent in winter wheat breeding. Molecular markers closely linked to the QTL can be used for marker-assisted selection. The identified QTL can help to develop cultivars with multiple-resistance-genes in combination that will hopefully provide a long lasting consistent level of disease resistance.

### Author contributions

Buerstmayr M: phenotyping of stripe rust and leaf rust experiments in Austria, SSR markers, map calculation, map validation, QTL analysis, manuscript writing.

Matiasch L: phenotyping of leaf rust experiments in Austria.

Mascher F: phenotyping of stripe rust experiments in Switzerland.

Vida G: phenotyping of leaf rust experiments in Hungary.

Ittu M: phenotyping of leaf rust experiments in Romaina.

Robert O: phenotyping of stripe rust experiments in France.

Holdgate S: phenotyping of stripe rust experiments in UK.

Flath K: providing of stripe rust spores.

Neumayer A: phenotyping of stripe rust and leaf rust experiments in Austria.

Buerstmayr H: project leader and project supervisor.

## Electronic supplementary material

Below is the link to the electronic supplementary material.

Supplementary material 1 (PDF 40 kb)

Supplementary material 2 (PDF 33 kb)

Supplementary material 3 (PDF 149 kb)

Supplementary material 4 (PDF 99 kb)

Supplementary material 5 (XLSX 22 kb)

Supplementary material 6 (PDF 91 kb)

Supplementary material 7 (PDF 28 kb)
