# Supplementary material for: Mapping of quantitative adult plant field resistance to leaf rust and stripe rust in two European winter wheat populations reveals co-location of three QTL conferring resistance to both rust pathogens
Source: Theor Appl Genet. 2014 Aug 12;127(9):2011–28. doi: 10.1007/s00122-014-2357-0 (PMC4145209; doi:10.1007/s00122-014-2357-0)
Supplement: Supplementary file 1 — Supplementary material 1 (PDF 40 kb) [file 122_2014_2357_MOESM1_ESM.pdf]

Online Resource 1

Article title: Mapping of quantitative adult plant field resistance to leaf rust and stripe rust in two European winter wheat populations reveals co-location of three QTL conferring resistance to both rust pathogens.

Journal: Theoretical and Applied Genetics

Authors: Maria Buerstmayr, Lydia Matiasch, Fabio Mascher, Gyula Vida, Marianna Ittu, Olivier Robert, Sarah Holdgate, Kerstin Flath, Anton Neumayer, Hermann Buerstmayr

Name, affiliation, and email of corresponding author:

Hermann Buerstmayr,
Department for Agrobiotechnology Tulln, BOKU-University
of Natural Resources and Life Sciences-Vienna,
Konrad Lorenz Str. 20, 3430 Tulln, Austria
e-mail: hermann.buerstmayr@boku.ac.at

Online resource 1: Detailed description of leaf rust experiments

population	year	country[1]	location	coordinates	experimental design	No. repl.	plot			spreader	
							No. of rows per RIL	length in m	spacing in cm	cultivars	arrangement
CA	2008	RO	Fundulea	44° 30′ N, 24° 10′ E	not random	2	single row	1.5	30	Ritmo+Arina	after each row
CA	2008	HU	Martonvásár	47°19'05" N, 18°46'26"E	not random	2	double row	1	20	Alcedo+Mv9kr1	after each double row
CA	2008	AT	Probstdorf	48° 00′ 10″ N, 16° 37′ 25″ E	RCB[2]	2	double row	1	17	Ritmo+Arina	after each double row
CA	2008	AT	Tulln	48° 20′ 0″ N, 16° 3′ 0″ E	RCB[2]	2	double row	1	17	Ritmo+Arina	after each double row
CA	2008	AT	Rust	48° 18′ 60″ N, 15° 55′ 23″ E	RCB[2]	2	double row	1	17	Ritmo+Arina	after each double row
CA	2008	AT	Schmida	48° 22′ 50″ N, 16° 08′ 00″ E	RCB[2]	2	double row	1	17	Ritmo+Arina	after each double row
CA	2009	AT	Rust	48° 18′ 60″ N, 15° 55′ 23″ E	RCB[2]	2	double row	1	17	Ritmo+Arina	after each double row
CA	2009	AT	Tulln	48° 20′ 0″ N, 16° 3′ 0″ E	RCB[2]	2	double row	1	17	Ritmo+Arina	after each double row
CF	2004	AT	Tulln	48° 20′ 0″ N, 16° 3′ 0″ E	RCB[2]	2	double row	1	17	Ritmo+Arina	after each double row
CF	2006	AT	Probstdorf	48° 00′ 10″ N, 16° 37′ 25″ E	RCB[2]	2	double row	1	17	Ritmo+Arina	after each double row
CF	2007	AT	Tulln	48° 20′ 0″ N, 16° 3′ 0″ E	RCB[2]	2	double row	1	17	Ritmo+Arina	after each double row
CF	2008	AT	Tulln	48° 20′ 0″ N, 16° 3′ 0″ E	RCB[2]	2	double row	1	17	Ritmo+Arina	after each double row

[1] Country abbreviated by the ISO 3166 country code, [2] RCB = randomized complete block design.

popu lation	year	location	inoculation technique	inoculation date	spores	scoring
CA	2008	Fundulea	dusting a mixture of spores: talcum on spreaders	8.4+21.4	collection of urediniospores from the previous year	% diseased leaf area
			planting infected seedlings into spreader rows	12.3+24.3+26.3	collection of urediniospores from the previous year	
CA	2008	Martonvásár	inoculation of spreaders by syringe (10 plants/m)	30.4	urediniospores multiplied in greenhouse	% diseased leaf area
CA	2008	Probstdorf	spray inoculation of spreaders	7.5+14.5	collection of urediniospores from the previous year	% diseased leaf area
CA	2008	Tulln	spray inoculation of spreaders	2.5+9.5+23.5	collection of urediniospores from the previous year	% diseased leaf area
CA	2008	Rust	spray inoculation of spreaders	5.5+8.5+23.5	collection of urediniospores from the previous year	% diseased leaf area
CA	2008	Schmida	spray inoculation of spreaders	6.5.+13.5+23.5	collection of urediniospores from the previous year	% diseased leaf area
CA	2009	Rust	spray inoculation of spreaders and lines + planting of infected seedlings into spreader rows	15.4+19.4	collection of urediniospores from the previous year	% diseased leaf area
CA	2009	Tulln		15.4+19.4	collection of urediniospores from the previous year	% diseased leaf area
CF	2004	Tulln	planting of infected seedlings into spreader rows	unknown	collection of urediniospores from the previous year	% diseased leaf area
CF	2006	Probstdorf	spray inoculation of spreaders	19.5+31.5	collection of urediniospores from the previous year	% diseased leaf area
CF	2007	Tulln	spray inoculation of spreaders + planting infected seedlings into spreaders	12.4+7.5<br>25.4+2.5	collection of urediniospores from the previous year	% diseased leaf area
CF	2008	Tulln	spray inoculation of spreaders	2.5+9.5+23.5	collection of urediniospores from the previous year	% diseased leaf area
