# Supplementary material for: Mapping of quantitative adult plant field resistance to leaf rust and stripe rust in two European winter wheat populations reveals co-location of three QTL conferring resistance to both rust pathogens
Source: Theor Appl Genet. 2014 Aug 12;127(9):2011–28. doi: 10.1007/s00122-014-2357-0 (PMC4145209; doi:10.1007/s00122-014-2357-0)
Supplement: Supplementary file 2 — Supplementary material 2 (PDF 33 kb) [file 122_2014_2357_MOESM2_ESM.pdf]

Online Resource 2

Article title: Mapping of quantitative adult plant field resistance to leaf rust and stripe rust in two European winter wheat populations reveals co-location of three QTL conferring resistance to both rust pathogens.

Journal: Theoretical and Applied Genetics

Authors: Maria Buerstmayr, Lydia Matiasch, Fabio Mascher, Gyula Vida, Marianna Ittu, Olivier Robert, Sarah Holdgate, Kerstin Flath, Anton Neumayer, Hermann Buerstmayr

Name, affiliation, and email of corresponding author:

Hermann Buerstmayr,
Department for Agrobiotechnology Tulln, BOKU-University
of Natural Resources and Life Sciences-Vienna,
Konrad Lorenz Str. 20, 3430 Tulln, Austria
e-mail: hermann.buerstmayr@boku.ac.at

Online Resource 2: Detailed description of stripe rust experiments

population		year	country[1]	location	coordinates	experimental design	No. Repl.	plot: No. rows per RIL	plot: length in m	plot: spacing in cm	spreader: cultivar	spreader: arrangement
CF	-	2009	AT	Reichersberg	48° 33′ 17″ N, 13° 34′ 60″ E	RCB[2]	2	3	1	19	no	------
CF	-	2010	AT	Reichersberg	48° 33′ 17″ N, 13° 34′ 60″ E	RCB[2]	2	3	1	19	no	------
CF	-	2010	AT	Tulln	48° 20′ 0″ N, 16° 3′ 0″ E	RCB[2]	2	2	1	17	Victo+Furore	after each double row
CF	CA	2011	AT	Tulln	48° 20′ 0″ N, 16° 3′ 0″ E	RCB[2]	2	2	1	17	Victo+Furore	after each double row
CF	CA	2012	AT	Atzenbrugg	48° 17′ 00″ N, 15° 52′ 27″ E	RCB[2]	2	2	1	17	Victo+Furore	after each double row
-	CA	2012	AT	Reichersberg	48° 33′ 17″ N, 13° 34′ 60″ E	RCB[2]	2	3	1	19	no	------
CF	CA	2012	CH	Changins	46° 23' 46'' N, 6° 14' 26'' E	RCB[2]	2	2	0.8	30	Eridano+Arbola+Coker	after every 2nd double row
-	CA	2012	FR	Cappelle en Pévèle	50° 31' 00"N; 3° 10' 00" E	RCB[2]	2	2	1	17	Toisondor	after every 5th double row
CF	CA	2012	GB	Cambridge	52° 14' 10"N; 0° 05 '32"E	RCB[2]	2	2	1	19	Vuka	
CF	CA	2012	GB	Ickleton	0° 16' 81"E; 52° 06 '08"E	RCB[2]	2	2	0.9	24	Vuka+AC Barrie	after every 2nd double row

[1] Country abbreviated by the ISO 3166 country code, [2] RCB = randomized complete block design.

population		year	location	inoculation technique	date of inoculation	plant growth stage	scoring method	used pathotype/pathotype mix	virulence profiles
CF	-	2009	Reichersberg	inoculation with microsprayer	17.04+29.04	EC29+EC32	% diseased leaf area	R41E168, R39E134, R6E22, R237E141, R32E0, R33E32, R44E204, R108E141, R109E141, R169E136, R232E137	v1,2,3,4,6,7,8,9,15,17,25, 27,32, Sd,Su,Sp,An,AvS, 18,Oakley
CF	-	2010	Reichersberg	inoculation with microsprayer	27.04+01.05	EC30+EC31	% diseased leaf area		
CF	-	2010	Tulln	inoculation with microsprayers + planting infected seedlings into spreaders	21.04+26.04	EC29+EC30	% diseased leaf area		
CF	CA	2011	Tulln	inoculation with microsprayers + planting infected seedlings into spreaders	23.04+02.05	EC29+EC30	% diseased leaf area		
CF	CA	2012	Atzenbrugg	inoculation with microsprayers + planting infected seedlings into spreaders	14.04+18.04	EC27+EC29	% diseased leaf area	R109E141, R237E141, Oakley, 20/11, 32/08, 19/09, 05/11, 10/09, 15/11	v1,2,3,4,5,6,7,8,9,10,17,24, 25,27,32, Sd,Su,Sp,An,AvS,So,18
-	CA	2012	Reichersberg	inoculation with microsprayer	20.04+24.04	EC27+EC29	1 – 9[a]		
CF	CA	2012	Changins	inoculation with microsprayers + planting infected seedlings into spreaders	05.03+12.04	EC27+EC29	% diseased leaf area	W474E412, W466E432, W396E300, incl. Yr17, CV	v1,2,3,3a,4,4a,6,7,8,9,11,17 (Audace and VPM1), 22,23,25,32,Sd,S/O,ND, CV1,CV2,CV3,Sp,Hv
-	CA	2012	Cappelle en Pévèle	planting infected seedlings into each spreaders	unknown	unknown	1 – 9[b]	239E175 V17=Warrior/Ambition	v1,2,3,4,6,7,9,17,25,32,Su
CF	CA	2012	Cambridge	natural epidemic	----	-----	1 – 5[c]	-----	-----
CF	CA	2012	Ickleton	planting infected seedlings into each spreaders	27.03	unknown	1 – 9[a]	08/21, 08/501, 03/7, 11/8	v1,2,3,4,7,9,17,32,Brock, Oakley,Robigus,Solstice, Timber,Warrior

[a] 1=0%, 2=1%,3=2%,4=5%,5=10%,6=25%,7=50%,8=75%,9=100%

[b] 1=0%, 2=10%, 3=20%, ………8=90%,9=100%

[c] 1=5%, 2=20%, 3=40%, 4=70%, 5=90%
