# Supplementary material for: Mapping of quantitative adult plant field resistance to leaf rust and stripe rust in two European winter wheat populations reveals co-location of three QTL conferring resistance to both rust pathogens
Source: Theor Appl Genet. 2014 Aug 12;127(9):2011–28. doi: 10.1007/s00122-014-2357-0 (PMC4145209; doi:10.1007/s00122-014-2357-0)
Supplement: Supplementary file 3 — Supplementary material 3 (PDF 149 kb) [file 122_2014_2357_MOESM3_ESM.pdf]

Online Resource 3

Article title: Mapping of quantitative adult plant field resistance to leaf rust and stripe rust in two European winter wheat populations reveals co-location of three QTL conferring resistance to both rust pathogens.

Journal: Theoretical and Applied Genetics

Authors: Maria Buerstmayr, Lydia Matiasch, Fabio Mascher, Gyula Vida, Marianna Ittu, Olivier Robert, Sarah Holdgate, Kerstin Flath, Anton Neumayer, Hermann Buerstmayr

Name, affiliation, and email of corresponding author:

Hermann Buerstmayr,
Department for Agrobiotechnology Tulln, BOKU-University
of Natural Resources and Life Sciences-Vienna,
Konrad Lorenz Str. 20, 3430 Tulln, Austria
e-mail: hermann.buerstmayr@boku.ac.at

Online resource 3 Spearman rank-correlation coefficients for percent infected leaf area of stripe rust and leaf rust experiments of population Capo × Arina and Capo × Furore

Spearman correlation coefficients for percent infected leaf area (YrS) between stripe rust experiments of population Capo × Arina

	2012 Atzenbrugg/AT		2012 Reichersberg/AT		2012 Cappelle/FR		2012 Changins/CH		2012 Cambridge/GB		2012 Ickleton/GB	
	r	*p*	r	*p*	r	*p*	r	*p*	r	*p*	r	*p*
2011 Tulln/AT	0.82	<.0001	0.68	<.0001	0.81	<.0001	0.74	<.0001	0.60	0.0005	0.83	<.0001
2012 Atzenbrugg/AT			0.73	<.0001	0.80	<.0001	0.76	<.0001	0.62	0.0002	0.75	<.0001
2012 Reichersberg/AT					0.67	<.0001	0.65	<.0001	0.74	<.0001	0.65	<.0001
2012 Cappelle/FR							0.81	<.0001	0.64	0.0001	0.82	<.0001
2012 Changins									0.58	0.0008	0.67	<.0001
2012 Cambridge/GB											0.65	0.0001

Spearman correlation coefficients for percent infected leaf area (YrS) between stripe rust experiments of population Capo × Furore

	2010 Tulln/AT		2010 Reichersberg/AT		2011 Tulln/AT		2012 Atzenbrugg/AT		2012 Changins/CH		2012 Camebridge/GB		2012 Ickleton/GB	
	r	*p*	r	*p*	r	*p*	r	*p*	r	*p*	r	*p*	r	*p*
2009 Reichersberg/AT	0.57	<.0001	0.80	<.0001	0.66	<.0001	0.76	<.0001	0.81	<.0001	0.89	<.0001	0.83	<.0001
2010 Tulln/AT			0.58	<.0001	0.69	<.0001	0.53	<.0001	0.72	<.0001	0.73	<.0001	0.71	<.0001
2010 Reichersberg/AT					0.70	<.0001	0.72	<.0001	0.81	<.0001	0.88	<.0001	0.79	<.0001
2011 Tulln/AT							0.67	<.0001	0.75	<.0001	0.69	<.0001	0.85	<.0001
2012 Atzenbrugg/AT									0.76	<.0001	0.86	<.0001	0.80	<.0001
2012 Changins											0.85	<.0001	0.83	<.0001
2012 Ickleton/GB													0.78	<.0001

Spearman correlation coefficients for percent infected leaf area (LrS) between leaf rust experiments of population Capo × Arina

	2008 Probstdorf/AT		2008 Fundulea/RO		2008 Rust/AT		2009 Rust/AT		2008 Tulln/AT		2009 Tulln/AT		2008 Marton/HU	
	r	*p*	r	*p*	r	*p*	r	*p*	r	*p*	r	*p*	r	*p*
2008 Schmida/AT	0.60	<.0001	0.52	<.0001	0.56	<.0001	0.68	<.0001	0.66	<.0001	0.64	<.0001	0.55	<.0001
2008 Probstdorf/AT			0.45	<.0001	0.52	<.0001	0.70	<.0001	0.57	<.0001	0.67	<.0001	0.52	<.0001
2008 Fundulea/RO					0.54	<.0001	0.60	<.0001	0.58	<.0001	0.61	<.0001	0.57	<.0001
2008 Rust/AT							0.67	<.0001	0.60	<.0001	0.69	<.0001	0.59	<.0001
2009 Rust/AT									0.71	<.0001	0.78	<.0001	0.63	<.0001
2008 Tulln/AT											0.72	<.0001	0.67	<.0001
2009 Tulln/AT													0.61	<.0001

Spearman correlation coefficients for percent infected leaf area (LrS) between leaf rust experiments of population Capo × Furore

	2007 Tulln/AT		2008 Tulln/AT		2004 Tulln/AT	
	r	*p*	r	*p*	r	*p*
2006 Probstdorf/AT	0.48	<.0001	0.73	<.0001	0.68	<.0001
2007 Tulln/AT			0.39	<.0001	0.40	<.0001
2008 Tulln/AT					0.65	<.0001
