# Supplementary material for: Mapping of quantitative adult plant field resistance to leaf rust and stripe rust in two European winter wheat populations reveals co-location of three QTL conferring resistance to both rust pathogens
Source: Theor Appl Genet. 2014 Aug 12;127(9):2011–28. doi: 10.1007/s00122-014-2357-0 (PMC4145209; doi:10.1007/s00122-014-2357-0)
Supplement: Supplementary file 4 — Supplementary material 4 (PDF 99 kb) [file 122_2014_2357_MOESM4_ESM.pdf]

Online Resource 4

Article title: Mapping of quantitative adult plant field resistance to leaf rust and stripe rust in two European winter wheat populations reveals co-location of three QTL conferring resistance to both rust pathogens.

Journal: Theoretical and Applied Genetics

Authors: Maria Buerstmayr, Lydia Matiasch, Fabio Mascher, Gyula Vida, Marianna Ittu, Olivier Robert, Sarah Holdgate, Kerstin Flath, Anton Neumayer, Hermann Buerstmayr

Name, affiliation, and email of corresponding author:

Hermann Buerstmayr,
Department for Agrobiotechnology Tulln, BOKU-University
of Natural Resources and Life Sciences-Vienna,
Konrad Lorenz Str. 20, 3430 Tulln, Austria
e-mail: hermann.buerstmayr@boku.ac.at

Online Resource 4 Analysis of variance for stripe rust and leaf rust severity measured by percentage of infected leaf area

	population Capo × Arina				population Capo × Furore			
Stripe rust								
Source of variation	*df*	Mean Square	*F*-value	*p*-value	*df*	Mean Square	*F*-value	*p*-value
Replication within exp	5	2.0	2.0	0.0844	6	12.9	10.7	<.0001
Experiment	4	181.8	177.8	<.0001	5	411.5	343.7	<.0001
Genotype	173	37.4	36.6	<.0001	202	34.8	29.1	<.0001
Genotype x Experiment	691	2.7	2.7	<.0001	941	2.6	2.1	<.0001
Error	867	1.0			1195	1.2		
Leaf rust								
Replication within exp	8	491	5	<.0001	5	15016	250.9	<.0001
Experiment	7	80753	814	<.0001	4	142109	2374.4	<.0001
Genotype	232	2953	30	<.0001	200	588	9.8	<.0001
Genotype x Experiment	1599	315	3	<.0001	799	108	1.8	<.0001
Error	1679	99			987	60		
