# Supplementary material for: Mapping of quantitative adult plant field resistance to leaf rust and stripe rust in two European winter wheat populations reveals co-location of three QTL conferring resistance to both rust pathogens
Source: Theor Appl Genet. 2014 Aug 12;127(9):2011–28. doi: 10.1007/s00122-014-2357-0 (PMC4145209; doi:10.1007/s00122-014-2357-0)
Supplement: Supplementary file 6 — Supplementary material 6 (PDF 91 kb) [file 122_2014_2357_MOESM6_ESM.pdf]

Online Resource 6

Article title: Mapping of quantitative adult plant field resistance to leaf rust and stripe rust in two European winter wheat populations reveals co-location of three QTL conferring resistance to both rust pathogens.

Journal: Theoretical and Applied Genetics

Authors: Maria Buerstmayr, Lydia Matiasch, Fabio Mascher, Gyula Vida, Marianna Ittu, Olivier Robert, Sarah Holdgate, Kerstin Flath, Anton Neumayer, Hermann Buerstmayr

Name, affiliation, and email of corresponding author:

Hermann Buerstmayr,
Department for Agrobiotechnology Tulln, BOKU-University
of Natural Resources and Life Sciences-Vienna,
Konrad Lorenz Str. 20, 3430 Tulln, Austria
e-mail: hermann.buerstmayr@boku.ac.at

Online Resource 6

Duncan multiple range test for mean of stripe rust severity (YrS) of subpopulation CF (31 RIL) tested at Cambridge/GB and Ickleton/GB. Classes were differentiated by number of resistance improving alleles at *QYr.ifa-2BL* and *QYr.ifa-3BS*

Number of QTL	Number of RIL	Mean	Std Dev	Duncan Grouping[a]
none	8	55.8	9.3	A
1	15	29.1	7.9	B
2	8	14.1	8.2	C

[a] means of QTL groups with different letters are significantly different at $p<0.05$

Duncan multiple range test for mean stripe rust severity (YrS) of subpopulation CA (31 RIL) tested at Cambridge/GB and Ickleton/GB. Classes were differentiated by number of resistance improving alleles at *QYr.ifa-2AL*, Q*Yr.ifa-2BL*, *QYr.ifa-2AS* and *QYr.ifa-3BS*

Number of QTL	Number of RIL	Mean	Std Dev	Duncan Grouping[a]
none	2	40.0	7.1	A
1	8	25.9	3.4	B
2	11	21.1	6.6	BC
3	8	16.6	3.5	C
4	2	17.5	3.5	C

[a] means of QTL groups with different letters are significantly different at $p<0.05$
