# Supplementary material for: Mapping of quantitative adult plant field resistance to leaf rust and stripe rust in two European winter wheat populations reveals co-location of three QTL conferring resistance to both rust pathogens
Source: Theor Appl Genet. 2014 Aug 12;127(9):2011–28. doi: 10.1007/s00122-014-2357-0 (PMC4145209; doi:10.1007/s00122-014-2357-0)
Supplement: Supplementary file 7 — Supplementary material 7 (PDF 28 kb) [file 122_2014_2357_MOESM7_ESM.pdf]

## Online Resource 7

**Article title:** Mapping of quantitative adult plant field resistance to leaf rust and stripe rust in two European winter wheat populations reveals co-location of three QTL conferring resistance to both rust pathogens.

**Journal:** Theoretical and Applied Genetics

**Authors:** Maria Buerstmayr, Lydia Matiasch, Fabio Mascher, Gyula Vida, Marianna Ittu, Olivier Robert, Sarah Holdgate, Kerstin Flath, Anton Neumayer, Hermann Buerstmayr

**Name, affiliation, and email of corresponding author:**

Hermann Buerstmayr,
Department for Agrobiotechnology Tulln, BOKU-University
of Natural Resources and Life Sciences-Vienna,
Konrad Lorenz Str. 20, 3430 Tulln, Austria
e-mail: hermann.buerstmayr@boku.ac.at

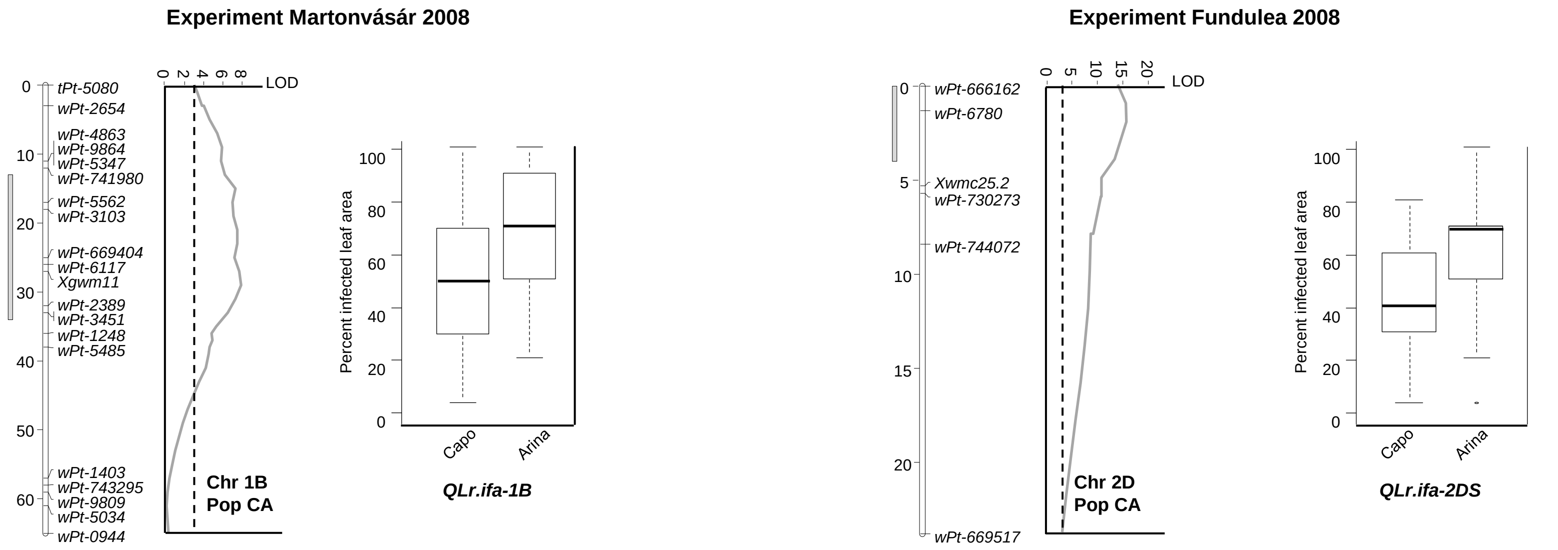


**Fig. S1** Linkage maps and positions of *QLr.ifa-1B* and *QLr.ifa-2DS* (QTL for leaf rust severity) determined by the MQM model. *QLr.ifa-1B* was identified in experiment at Martonvásár 2008 and *QLr.ifa-2DS* was identified in experiment at Fundulea 2008. LOD profiles are given on the right; bars of the QTL support interval are given on the left. Bar size indicates a LOD decrease of 1.5 from maximum LOD. The dashed lines represent the LOD 3 value. Boxplot distributions of lines possessing alternative alleles at *QLr.ifa-1B* and *QLr.ifa-2DS* measured in percentage of infected leaf area are shown. Genotypes were classified by allele status of the closest markers to the corresponding QTL. Data are based on the means of experiment conducted at Martonvásár 2008 and at Fundulea 2008, respectively. Medians are indicated by solid lines
